# Supplementary material for: Generation of a Sleeping Beauty Transposon-Based Cellular System for Rapid and Sensitive Screening for Compounds and Cellular Factors Limiting SARS-CoV-2 Replication
Source: Front Microbiol. 2021 Jul 29;12:701198. doi: 10.3389/fmicb.2021.701198 (PMC8362758; doi:10.3389/fmicb.2021.701198)
Supplement: Supplementary file 6 [file Image_1.pdf]

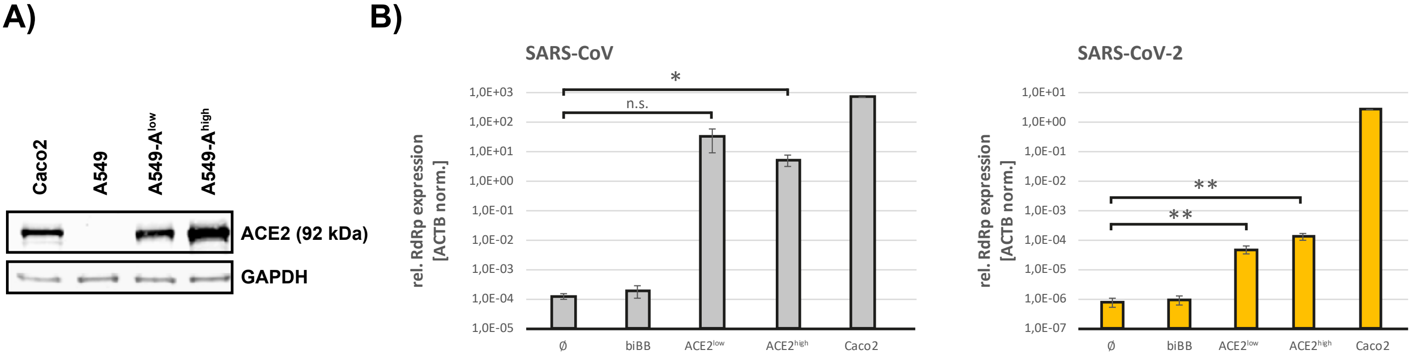

Supplementary Figure 1 - Expression of the ACE2 receptor renders A549 cells permissive for SARS-CoV and to a lesser extent to SARS-CoV-2. A) Western blot analysis of ACE2 in Caco2, parental A549 and ACE2 low (A549-A^{low}) and high (A549-A^{high}) expressing cells. GAPDH was used for loading control. **B)** RT-qPCR analysis of intracellular RNA obtained from SARS-CoV (left) and SARS-CoV-2 (right) infected A549 cells and derivates as indicated. Relative expression of SARS-CoV-2 RNA targeting RdRp-gene. Values were normalized to ACTB expression. Error bars indicate SD from the mean of representative experiment performed in triplicates (except representative result for Caco2 cells, n=1).

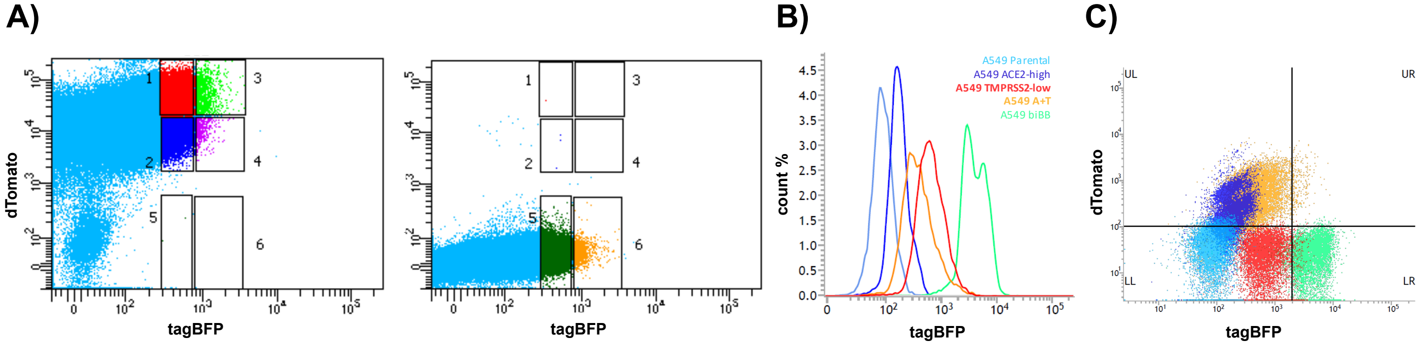

Supplementary Figure 2 - Gating strategy and cell sorting of A549-AT cells. A) A549 cells co-transfected with ACE2 and TMPRSS2 were sorted based on their constitutive dTomato and tagBFP intensities which served as a surrogate marker for expression of ACE2 and TMPRSS2, respectively (left scatter plot). Population 1: A549-ACE2^{veryhigh}+TMPRSS2^{low}, Population 2: A549-ACE2^{high}+TMPRSS2^{high}, Population 3: A549-ACE2^{veryhigh}+TMPRSS2^{high}, Population 4: A549-ACE2^{high} + TMPRSS2^{high}. Population 1 and 2 were gated to obtain cells with low tagBFP levels paired with high and very high dTomato levels. High tagBFP levels in population 3 and 4 were combined with high and very high dTomato levels. Moreover, A549 were transfected with TMPRSS2 (right scatter plot) and gates for tagBFP set accordingly to population 1-4. In subsequent infection experiments, Population 2 demonstrated the most distinct CPE and was therefore assigned as the model cell line “A549-AT”. **B-C)** 2 weeks post sorting, dTomato and tag-BFP levels were confirmed by flow cytometry. A549-AT cells (orange) constitute dTomato iintensities comparable to A549-A cells (dark blue) and tagBFP intensities nearly identical to A549-TMPRSS2-low cells (red). Parental A549 cells (light blue) and sorted A549 cells expressing Luciferase and very high tagBFP levels (biBB in green) are shown for more accurate estimation of fluorescence intensities.

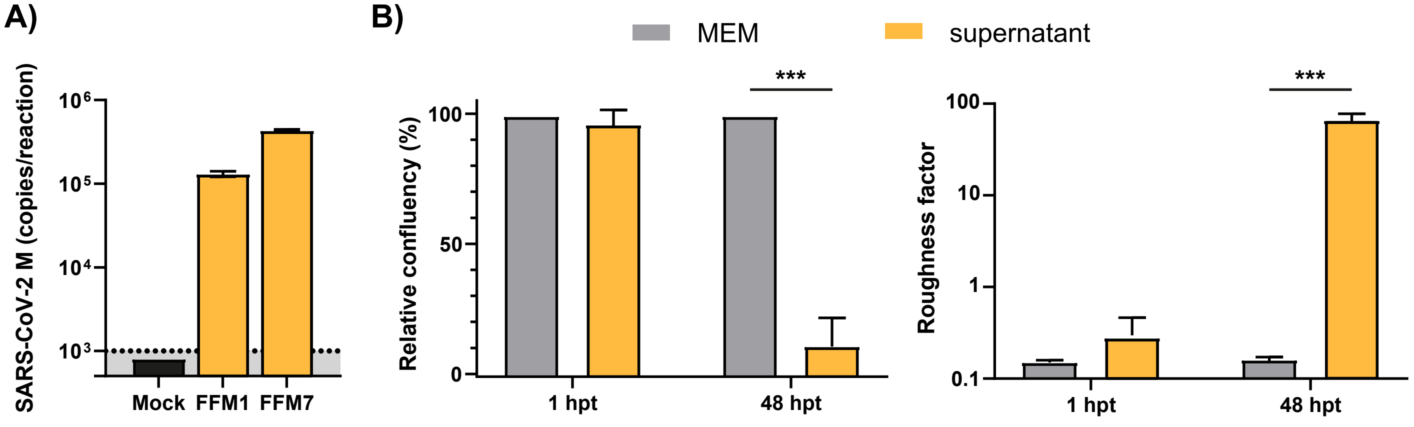

Supplementary Figure 3 - The supernatant of SARS-CoV-2 infected A549-AT cells contains infectious and replication-competent virus.
A) RT-qPCR analysis of RNA obtained from the supernatant of SARS-CoV-2 infected A549-AT cells (24 h post infection, MOI = 0.1) targeting M-Gene (Toptan et al. 2020). **B)** Relative confluency (left) and roughness factor (right) of A549-AT cells infected with cell culture supernatant from SARS-CoV-2 infected A549-AT cells (72 h post infection) . Error bars indicate SD from the mean of representative experiments performed in triplicates (A) or quadruplicate (B).
